# Supplementary material for: Gene silencing in plants by artificial small RNAs derived from minimal precursors and expressed via tobacco rattle virus
Source: Plant Mol Biol. 2025 Nov 27;115(6):131. doi: 10.1007/s11103-025-01661-y (PMC12660477; doi:10.1007/s11103-025-01661-y)
Supplement: Supplementary file 3 — Supplementary file3 (PDF 1849 KB) [file 11103_2025_1661_MOESM3_ESM.pdf]

SUPPLEMENTARY INFORMATION

Data S1. sRNA reads from art-sRNA-expressing tissues.

Data S2. 21-nt siRNAs from *NbSu* and *NbTAS3*.

Figure S1. Photos at 28 days post-inoculation (dpi) of sets of three plants agroinoculated with the different constructs and inoculated or not (mock) with TSWV.

Figure S2. Two-dimensional line graph showing, for each of the 16-plant sets listed, the percentage of symptomatic plants per day during 28 days.

Figure S3. Phasing analysis of syn-tasiRNA target RNA-derived 21 nucleotide small RNAs. Radar plots show proportions of 21-nucleotide reads corresponding to each of the 21 registers from Su, with position 1 designated as immediately after the syn-tasiRNA-guided cleavage site. Control plots for TAS3 are shown. The percentage of 21-nucleotide reads corresponding to phasing register 1 is indicated.

Table S1. Name, sequence and use of DNA oligonucleotides used in this study.

Text S1. Protocol to generate TRV-based art-sRNA constructs.

Text S2. DNA sequence in FASTA format of all precursors used to express art-sRNAs in plants.

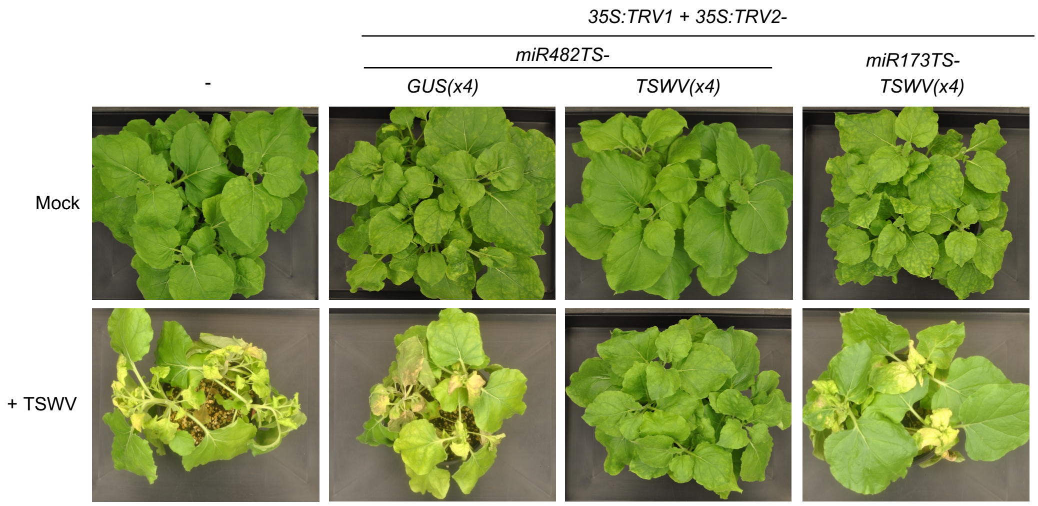

Figure S1. Photos at 28 days post-inoculation (dpi) of sets of three plants agroinoculated with the different constructs and inoculated or not (mock) with TSWV.

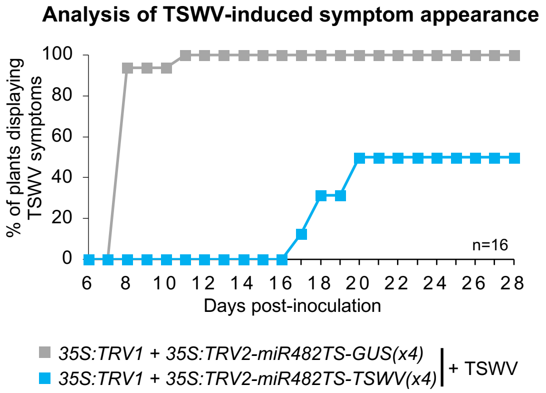

Figure S2. Two-dimensional line graph showing, for each of the 16-plant sets listed, the percentage of symptomatic plants per day during 28 days.

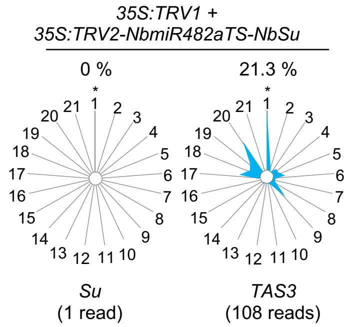

Figure S3. Phasing analysis of syn-tasiRNA target RNA-derived 21 nucleotide small RNAs. Radar plots show proportions of 21-nucleotide reads corresponding to each of the 21 registers from *Su*, with position 1 designated as immediately after the syn-tasiRNA-guided cleavage site. Control plots for *TAS3* are shown. The percentage of 21-nucleotide reads corresponding to phasing register 1 is indicated.

Table S1. Name, sequence and use of DNA oligonucleotides used in this study.

Name	Sequence	Type*	Construct/Aim
AC-50	CCGATTCACCCAGAGCTGATA	ssDNA	Probe to detect syn-tasiR-TSWV-2
AC-51	CGGTATGCCCCACTATACCAA	ssDNA	Probe to detect syn-tasiR-TSWV-3
AC-52	AAGATGGATTGTGCACTCTGA	ssDNA	Probe to detect syn-tasiR-TSWV-4
AC-55	AGGGGCCATGCTAATCTTCTC	ssDNA	Probe for U6 detection
AC-355	GACCCTGATGTTGATGTTCGCT	ssDNA	qPCR amplification of *Su* mRNA
AC-356	GAGGGATTTGAAGAGAGATTTC	ssDNA	
AC-365	GACCCTGATGTTGATGTTCGCT	ssDNA	PCR&qPCR amplification of *PP2A* mRNA
AC-366	GAGGGATTTGAAGAGAGATTTC	ssDNA	
AC-416	A+GGA+CAC+AAT+CAC+GTC+TTA+CA	ssLNA	Probe to detect syn-tasiR-TSWV-1
AC-417	G+CGG+GAA+GTC+CAC+CAC+GGT+TA	ssLNA	Probe for amiR-Su/syn-tasiR-Su detection
AC-518	cacttacccgagttaacgccAAACCTAAACCTAAACGG	ssDNA	*35S:TRV2-TAS1c-miR482TS-Su*
AC-519	gtttaatgtcttcgggacatATTTCACTTTACGATGTGG	ssDNA	
AC-523	TCGGTTTGCTGACCTACTGG	ssDNA	art-sRNA precursor detection
AC-524	AACCTAAAACTTCAGACACGG	ssDNA	
AC-615	cacttacccgagttaacgccTATAGGGGGGAAAAAAAGGTAG	ssDNA	*35S:TRV2-pri-amiR-Su*
AC-616	gtttaatgtcttcgggacatGAGACTAAAGATGAGATCTAATCTG	ssDNA	
AC-617	cacttacccgagttaacgccAGTAGAGAAGAATCTGTA	ssDNA	*35S:TRV2-shc-amiR-Su*
AC-618	gtttaatgtcttcgggacatAGTAAGAAGAGCCAA	ssDNA	
AC-660	ATGGGAGATATGTACGATGAAT	ssDNA	TRV diagnostic
AC-661	GGGATTAGGACGTATCGGACC	ssDNA	
AC-667	cacttacccgagttaacgccGTGGTATGGGGGGAGTCGGGAATAGACCATTTATGTATGACTCCCGGAATTCCAatgtcccgaagacattaaac	dsDNA	*35S:TRV2-miR482TS-Su*
AC-984	cacttacccgagttaacgccGTGGTATGGGGGGAGTCGGGAATAGACCATTTATATTGACCCACACTTTGCCGATAACCTTCACCCGGTTGCCACTATTGACCCACACTTTGCCGATAACCTTCACCCGGTTGCCACatgtcccgaagacattaaac	dsDNA	*35S:TRV2-miR482TS-GUS(x4)*
AC-985	cacttacccgagttaacgccGTGGTATGGGGGGAGTCGGGAA	ssDNA	*35S-TRV2-miR482TS-TSWV(x4)*
AC-986	gtttaatgtcttcgggacatAAGATGGATTGTGCACTCTGA	ssDNA	
AC-1222	cacttacccgagttaacgccGTGATTTTTCTCTACAAGCGAA	ssDNA	*35S:TRV2-miR173TS-Su*
AC-1223	gtttaatgtcttcgggacatTGGAATTCCGGGAGTCATACATAAA	ssDNA	

*ssDNA: single-stranded DNA; dsDNA: double-stranded DNA; LNA: locked nucleic acid.

Text S1. Protocol to generate TRV-based art-sRNA constructs.

1. Preparation of the dsDNA art-sRNA insert

1.1. amiRNA insert

Design and order a dsDNA (eg. ultramer duplex in IDT) including the sequence of your amiRNA included in the *MIR390*-based ("*shc*") minimal precursor, as follows:

cacttacccgagttaacgccAGTAGAGAAGAATCTGTA$X_1X_2X_3X_4X_5X_6X_7X_8X_9X_{10}X_{11}X_{12}X_{13}X_{14}X_{15}X_{16}X_{17}X_{18}X_{19}X_{20}X_{21}$**AGTAGAGAAGAATC<u>TGTA</u>**$X_1X_2X_3X_4X_5X_6X_7X_8X_9X_{10}X_{11}X_{12}X_{13}X_{14}X_{15}X_{16}X_{17}X_{18}X_{19}X_{20}X_{21}$CGAAATCAAACT$X_1X_2X_1X_2X_3X_4X_5X_6X_7X_8X_9X_{10}X_{11}X_{12}X_{13}X_{14}X_{15}X_{16}X_{17}X_{18}X_{19}$CA**<u>TT</u>GGCTCTTCTTACT**atgtcccgaagacattaaac

Where:

-**X** is a DNA base of the amiRNA sequence, and the subscript number is the base position in the amiRNA 21-mer

-**X** is a DNA base of the amiRNA* sequence, and the subscript number is the base position in the amiRNA* 21-mer

-**X** is a DNA base of the basal stem (BS) region of the *shc* precursor

-X is a DNA base of the *shc* precursor included in the oligonucleotides required to clone the amiRNA insert in B/c vectors

-x is a DNA base of the TRV sequence, required for Gibson-based assembly

-<u>X</u> is a DNA base of the *shc* precursor included in the oligonucleotides required to clone the amiRNA insert in B/c vectors

-**X** is a DNA base of the *shc* precursor that may be modified to preserve the authentic *shc* duplex structure

In the sequence above:

-Insert the amiRNA sequence where you see $X_1X_2X_3X_4X_5X_6X_7X_8X_9X_{10}X_{11}X_{12}X_{13}X_{14}X_{15}X_{16}X_{17}X_{18}X_{19}X_{20}X_{21}$

-Insert the amiRNA* sequence that has to verify the following base-pairing:

X_1 X_2 X_3 X_4 X_5 X_6 X_7 X_8 X_9 $X_{10}X_{11}X_{12}X_{13}X_{14}X_{15}X_{16}X_{17}X_{18}$ X_{19} $X_{20}X_{21}$

| | | | | | | | | | | | | | | | | | | | |

$X_{19}X_{18}X_{17}X_{16}X_{15}X_{14}X_{13}X_{12}X_{11}X_{10}X_9$ X_8 X_7 X_6 X_5 X_4 X_3 X_2 X_1 X_2 X_1

Note that:

-In general, X_1=T for amiRNA association with AGO1. In this case, X_{19}=A

-Bases X_{11} and X_9 DO NOT base-pair to preserve the central bulge of the authentic *AtMIR390a* duplex. The following base-pair rule applies:

- -If X_{11}=G, then X_9=A
- -If X_{11}=C, then X_9=T
- -If X_{11}=A, then X_9=G
- -If X_{11}=U, then X_9=C

Fragment #1 (amiRNA precursor) is ready.

1.2. syn-tasiRNA insert

Design and order a dsDNA (eg. ultramer duplex in IDT) including the sequences of your syn-tasiRNA(s) (2 in the following example) following the 22-nt miRNA target site of interest, as follows:

agaggtcagcaccagctagc$X_1X_2X_3X_4X_5X_6X_7X_8X_9X_{10}X_{11}X_{12}X_{13}X_{14}X_{15}X_{16}X_{17}X_{18}X_{19}X_{20}X_{21}X_{22}$TAGA CCATTTA$X_1X_2X_3X_4X_5X_6X_7X_8X_9X_{10}X_{11}X_{12}X_{13}X_{14}X_{15}X_{16}X_{17}X_{18}X_{19}X_{20}X_{21}X_1X_2X_3X_4X_5X_6X_7X_8X_9X_{10}X_{11}$ $X_{12}X_{13}X_{14}X_{15}X_{16}X_{17}X_{18}X_{19}X_{20}X_{21}$agggtttgttaagtttccct

Where:

- -X is a DNA base of the 22-nt miRNA target site sequence, and the subscript number is the base position
- -X is a DNA base of the syn-tasiRNA-1 sequence, and the subscript number is the base position in the syn-tasiRNA* 21-mer
- -X is a DNA base of the syn-tasiRNA-2 sequence, and the subscript number is the base position in the syn-tasiRNA 21-mer
- -x is a DNA base of the TRV sequence, required for Gibson-based assembly
- -X is a DNA base of the *AtTAS1c* sequence

Note that:

- -In general, X_1=T and X_1=T for amiRNA association with AGO1.

Fragment #1 (syn-tasiRNA precursor) is ready.

2. Preparation of the vector

-Digest *pLB-PVX* with *Mlu*I.

-Gel purify the 9921 bp band corresponding to linearized plasmid.

-Quantify 1 ul in Nanodrop.

Fragment #2 (backbone vector) is ready.

3. Assembly

-Assemble the Gibbson reaction as described below:

Fragment 1 (dsDNA insert)[a]	
Fragment 2 (vector)[b,c,d]	
GeneArt Gibson Assembly HiFI Master Mix	5 µL
dH_2O	to 10 µL
Total volume	10 µL

[a]The optimal amount of vector is between 50-100 ng

[b]Insert/vector molar excess is between 2-3.

[c]Total DNA amount is between 0.02-0.5 pmol

[d]Mass to moles conversions can be calculated here: http://nebiocalculator.neb.com/#!/ssdnaamt

-Incubate reactions at 50ºC for 1h.

-Clean up reactions with a column (e.g. Zymo Research)

-Transform 1-4 µL in *E. coli* DH5α

-Plate in L-Kan plates and incubate 16h at 37ºC

4. Clone verification

-Pick several colonies and grow in liquid LB-Kan 16h at 37ºC, and purify plasmids.

-Digest candidate clones with *BpiI*

Good clones:

1 amiRNA: 2296 + 1064 + **1011 +** 1064 + 728 + 423 + 378 +3 bp

2 syn-tasiRNAs: 2296 **+** 1064 + **997** + 728 + 423 + 378 +3 bp

Bad clones (empty *pLX-TRV2*): 2296 + **1351 +** 1064 + 728 + 423 + 378 +3 bp

-Confirm insert sequence by Sanger sequencing with forward and reverse oligos AC-523 (`TCGGTTTGCTGACCTACTGG`) and/or AC-524 (`AACCTAAAACTTCAGACACGG`), respectively.

Text S2. DNA sequence in FASTA format of all precursors used to express art-sRNAs in plants.

1. amiRNA precursors

>pri-amiR-Su
TATAGGGGGGAAAAAAAGGTAGTCATCAGATATATATTTTGGTAAGAAAATATAGAAATGAATAATTTCACGTTTAACGAA
GAGGAGATGACGTGTGTTCCTTCGAACCCGAGTTTTGTTCGTCTATAAATAGCACCTTCTCTTCTCCTTCTTCCTCACTTC
CATCTTTTTAGCTTCACTATCTCTCTATAATCGGTTTTATCTTTCTCTAAGTCACAACCCAAAAAAACAAAGTAGAGAAGA
ATCTGTATGTATGACTCCCGGAATTCCAATGATGATCACATTCGTTATCTATTTTTTTGGAATTCCGTGAGTCATACACAT
TGGCTCTTCTTACTACAATGAAAAAGGCCGAGGCAAAACGCCTAAAATCACTTGAGAATCAATTCTTTTTACTGTCCATTT
AAGCTATCTTTTATAAACGTGTCTTATTTTCTATCTCTTTTGTTTAAACTAAGAAACTATAGTATTTTGTCTAAAACAAAA
CATGAAAGAACAGATTAGATCTCATCTTTAGTCTC

AtMIR390a
amiR-Su
amiR-Su*

>shc-amiR-Su
AGTAGAGAAGAATCTGTATGTATGACTCCCGGAATTCCACGAAATCAAACTTGGAATTCCGTGAGTCATACACATTGGCTC
TTCTTACT

AtMIR390a
OsMIR390
amiR-Su
amiR-Su*

2. syn-tasiRNA precursors

>TAS1c-Su
AAACCTAAACCTAAACGGCTAAGCCCGACGTCAAATACCAAAAAGAGAAAAACAAGAGCGCCGTCAAGCTCTGCAAATACGATCTGTAAG
TCCATCTTAACACAAAAGTGAGATGGGTTCTTAGATCATGTTCCGCCGTTAGATCGAGTCATGGTCTTGTCTCATAGAAAGGTACTTTCG
TTTACTTCTTTTGAGTATCGAGTAGAGCGTCGTCTATAGTTAGTTTGAGATTGCGTTTGTCAGAAGTTAGGTTCAATGTCCCGGTCCAAT
TTTCACCAGCCATGTGTCAGTTTCGTTCCTTCCCGTCCTCTTCTTTGATTTCGTTGGGTTACGGATGTTTTCGAGATGAAACAGCATTGT
TTTGTTGTGATTTTTCTCTACAAGCGAATAGACCATTTATGTATGACTCCCGGAATTCCATCGGTGGATCTTAGAAAATTATTCTAAGTC
CAACATAGCGTATTCTAAGTTCAACATATCGACGAACTAGAAAAGACATTGGACATATTCCAGGATATGCAAAAGAAAACAATGAATATT
GTTTTGAATGTGTTCAAGTAAATGAGATTTTCAAGTCGTCTAAAGAACAGTTGCTAATACAGTTACTTATTTCAATAAATAATTGGTTCT
AATAATACAAAACATATTCGAGGATATGCAGAAAAAAAAGATGTTTGTTATTTTGAAAAGCTTGAGTAGTTTCTCTCCGAGGTGTAGCGAA
GAAGCATCATCTACTTTGTAATGTAATTTTCTTTATGTTTTCACTTTGTAATTTTATTTGTGTTAATGTACCATGGCCGATATCGGTTTT
ATTGAAAGAAAATTTATGTTACTTCTGTTTTGGCTTTGCAATCAGTTATGCTAGTTTTCTTATACCCTTTCGTAAGCTTCCTAAGGAATC
GTTCATTGATTTCCACTGCTTCATTGTATATTAAAACTTTACAACTGTATCGACCATCATATAATTCTGGGTCAAGAGATGAAAATAGAA
CACCACATCGTAAAGTGAAAT

TAS1c
miR173a TS
syn-tasiR-Su

>miR482aTS-NbSu
GTGGTATGGGGGGAGTCGGGAATAGACCATTTATGTATGACTCCCGGAATTCCA

TAS1c
miR482 TS
syn-tasiR-Su

>miR173TS-Su
GTGATTTTTCTCTACAAGCGAATAGACCATTTATGTATGACTCCCGGAATTCCA

TAS1c
miR173 TS
syn-tasiR-NbSu

>miR482TS-GUS(x4)
GTGGTATGGGGGGAGTCGGGAATAGACCATTTATATTGACCCACACTTTGCCGATAACCTTCACCCGGTTGCCACTATTGACCC
ACACTTTGCCGATAACCTTCACCCGGTTGCCAC

AtTAS1c
NbmiR482a TS
syn-tasiR-GUS$_{Nb}$-1
syn-tasiR-GUS$_{Nb}$-2

>miR482TS-TSWV(x4)
GTGGTATGGGGGGAGTCGGGAATAGACCATTTATGTAAGACGTGATTGTGTCCTTATCAGCTCTGGGTGAATCGGTTGGTATAG
TGGGGCATACCGTCAGAGTGCACAATCCATCTT

AtTAS1c
miR482 TS
syn-tasiR-TSWV-1
syn-tasiR-TSWV-2
syn-tasiR-TSWV-3
syn-tasiR-TSWV-4

>miR173TS-TSWV(x4)
GTGATTTTTCTCTACAAGCGAATAGACCATTTATGTAAGACGTGATTGTGTCCTTATCAGCTCTGGGTGAATCGGTTGGTATAG
TGGGGCATACCGTCAGAGTGCACAATCCATCTT

TAS1c
miR173 TS
syn-tasiR-TSWV-1
syn-tasiR-TSWV-2
syn-tasiR-TSWV-3
syn-tasiR-TSWV-4
